# The Preparation of Dense Materials in the MgO–ZrO_2_ System by the Application of Nanometric Powders

**DOI:** 10.3390/ma14195478

**Published:** 2021-09-22

**Authors:** Kamil Wojteczko, Krzysztof Haberko, Katarzyna Berent, Paweł Rutkowski, Mirosław M. Bućko, Zbigniew Pędzich

**Affiliations:** 1 Faculty of Materials Science and Ceramics, AGH University of Science and Technology, 30-059 Kraków, Poland; haberko@agh.edu.pl (K.H.); pawelr@agh.edu.pl (P.R.); bucko@agh.edu.pl (M.M.B.); pedzich@agh.edu.pl (Z.P.); 2 Academic Centre for Materials and Nanotechnology, AGH University of Science and Technology, 30-059 Kraków, Poland; kberent@agh.edu.pl

**Keywords:** ZrO_2_, MgO, solid-state reaction

## Abstract

Crystallization under hydrothermal conditions allowed us to prepare nanometric powders in the MgO–ZrO_2_ system of different magnesia concentrations. Sintering runs of these powder compacts studied using dilatometry measurements during heating and cooling revealed essential differences in their behavior. The microstructure of the resulting polycrystal is strongly related to the magnesia content in the starting powder, which strongly influences the phase composition of the resulting material and its mechanical properties. It should be emphasized that the novel processing method of such materials differs from the usual applied technology and leads to magnesia–zirconia materials of a different microstructure than that of “classical” materials of this kind.

## 1. Introduction

Materials in the MgO–ZrO_2_ system were extensively studied in the 1980s–1990s. This was due to the potential ability of creating polycrystals with interesting mechanical properties, i.e., a high fracture toughness and a relatively high strength, through this system. A high refractoriness, a resistance to thermal shock conditions, and a resistance to attacks from metallurgical slags were also important. 

The concentrations of magnesia usually applied in the mentioned materials changed in the range of 8.1 to 9.7 mol%. Starting powders were synthetized via a solid-state reaction followed by grinding. Alternatively, mixtures of the constituent powders were compacted, and the solid state was formed directly during sintering at temperatures of 1700 or even 1850 °C [1,2,3,4,5,6,7]. According to the phase diagram of the system [8], these high processing temperatures with the indicated chemical composition of the system resulted in the MgO–ZrO_2_ solid solution of cubic symmetry. The temperature was then reduced to about 1400 °C, i.e., close to the eutectoid temperature level or even lower. This shifted the system to the two-phase field of the coexistence of the tetragonal and cubic symmetry of the MgO–ZrO_2_ solid solution. Annealing under such conditions resulted in the formation of the tetragonal symmetry zirconia inclusions within cubic grains. These phases have a definite crystallographic orientation, while such coherent interphase boundaries show low surface energy. Due to this, tetragonal-phase inclusions can be retained up to room temperature. This phenomenon is responsible for the increased fracture toughness of the material, due to the martensitic transformation of the tetragonal-phase inclusions to the monoclinic ones at the crack tip advancing through the material [9]. This transformation consumes strain energy, which could otherwise be used for crack propagation. 

The mechanical properties of the materials in the MgO–ZrO_2_ system prepared by this “classical” method, including at elevated temperatures, have been the subject of several papers [10,11,12,13]. Fewer studies have been devoted to the preparation and application of nanopowders in this system [14,15,16,17]. These are based on co-precipitation using ammonia and most probably alkalized ammonium chloride as a precipitating agent. Precipitated oxalate gels from the aqueous solutions of the relative salt solutions have also been used.

The main advantage of nanopowders is their high sintering ability; however, this is limited due to the presence of strong agglomerates, which lead to the essential difference in size between inter- and intra-agglomerate space and the poor densification of powder compacts. According to the statistical Rumpf [18] calculations, the tensile strength (P_c_) of agglomerates is given by the equation:

$$P_{c}\;=\;\frac{9}{8}\;\frac{1-V_{p}}{\pi d^{2}}\;L_{k}F_{k}$$

where V_p_ is the pore volume fraction within an agglomerate, L_k_ is the mean number of contacts per particle (crystallite) within an agglomerate, F_k_ is the force necessary to separate two particles in contact, and d is the size of a particle (crystallite). 

In order to decrease the strength of the nanometric powder agglomerates and produce a more uniform powder compaction, the co-precipitated powder precursor gels were washed with ethanol [14,16,17]. The resulting powder compacts showed a relatively uniform microstructure [16]. In order to produce the final crystalline powders, the authors of the referred studies [14,15,16,17] subjected the amorphous precursors to calcination at elevated temperatures, which led to the formation of strong bonds between the crystallites. 

A further decrease in the strength of the agglomerates can be attained by reducing the F_k_ factor in the Rumpf equation. The aim of the present investigation was to synthesize MgO–ZrO_2_ polycrystals of two different compositions through the application of powders of nanometric crystallite sizes. Such fine powders cannot be prepared via mechanical comminution. However, according to our previous investigations, the crystallization of zirconia and zirconia solid solutions under hydrothermal conditions leads to powders characterized by a narrow particle size distribution. More importantly, since crystallization occurs in a liquid medium, i.e., in water, which wets and penetrates between oxide particles, the formation of strong bonds between them does not occur [19], and they show an extremely high sintering ability [20,21]. The powder characteristics and the behavior of powder compacts during sintering, as well as some of the properties of the resulting materials, are shown in this paper.

## 2. Materials and Methods

Powders of two different compositions were prepared. The starting solutions of ZrOCl_2_ (2.1 M concentration) and proper amounts of MgCl_2_ were used. The one solution recalculated to oxides corresponded to 9 mol% MgO and the other one to 15 mol% MgO. Magnesium chloride was prepared via the dissolution of the analytical quality MgCO_3_ in hydrochloric acid. The main “impurity” (hafnium) in zirconium oxychloride recalculated to oxides corresponded to a HfO_2_ concentration of 1.68 wt%. An aqueous ammonia solution (4 M) was used as the precipitation agent. The mutual solutions of ZrOCl_2_ and MgCl_2_ were slowly introduced (100 cm^3^/min) to the vigorously stirred NH_3_ solution, which resulted in the co-precipitated X-ray amorphous gel. A final pH of about 10 was found. Polyethylene containers were used throughout the precipitation process to avoid possible silica contamination.

Our preliminary experiments indicated the presence of some Mg in the filtrate. Washing the precipitate with water removed about 40% of Mg introduced to the system. This is why the co-precipitated gel should not be washed with water. It is worth noting that this was not taken into account in the studies in [14,16,17] during the precipitation of the nanopowders in the MgO–ZrO_2_ system. This is why the control of Mg in the final product is necessary. In the present work, the co-precipitated gel was not washed, and the whole suspension in the mother liquor was then subjected to hydrothermal treatment (Parr equipment, type 4838) at 240 °C for 4 h with a rate of temperature increase of 5 °C/min. The process was performed in a Teflon vessel. The resulting material was then washed with distilled water until no reaction for the Cl^−^ ions (with AgNO_3_) in the filtrate occurred. The powder suspensions were then frozen by introducing them to liquid nitrogen and then freeze-dried using SRK System Technique (mod. GT2 Basic) equipment (SRK Systemtechnik GmbH, Riedstadt, Germany). The powders treated by this method are characterized by extremely soft agglomerates [20,21]. This helps to achieve a uniform powder compaction, which is especially important in the case of nanometric powders and is beneficial for the sintering of such powder compacts. 

The powders were characterized by measuring their specific surface area via nitrogen adsorption (BET) and pore size distribution in the powder compacts through the capillary condensation method (BJH) using Micromeritics equipment (Asap 2000) (Micromeritics, Norcross, Georgia, USA). X-ray diffraction analysis (Empyrean PANalytical, X’Pert HighScore Plus v3.05, Malvern Panalytical, Malvern, United Kingdom) allowed us to determine the phase composition of the powders via the Rietveld method and the powder crystallite sizes on the basis of the X-ray (111) line broadening of the cubic/tetragonal phase using the Scherrer formula and the proper correction due to instrument broadening. CuK_α1_ radiation was applied. Observations under a transmission electron microscope (FEI Tecnai FEG, 200 kV, Thermo Fisher Scientific, Hillsboro, OH, USA) were also useful. The concentration of MgO in the powders was determined using a wavelength dispersive X-ray fluorescence spectrometer (WDXRF Axios Fast mAX spectrometer, Rh-anode, maximum power 4 kW, Malvern Panalytical, Malvern, UK). Qualitative spectrum analysis was performed by identifying the spectral lines. The quantitative analysis was performed using the fundamental parameter method in the field of fluorine-uranium (F-U). The PANalytical standard-less analysis database package (Omnian) was used. The contents of the determined elements were normalized to 100% by mass.

The pelleting technique was completed with the addition of a cellulose-based binder. Sample ratio: binder = 2:1.

Uniaxial pressing (50 MPa) followed by cold isostatic repressing under 250 MPa was applied in order to prepare cylindrical samples of 20 mm in diameter and about 3 mm in thickness. They were used to observe shrinkage vs. temperature up to 1450 °C with a rate of temperature increase and cooling of 5 °C/min. The dilatometer Netzsch DIL 402C (Erich NETZSCH GmbH & Co. Holding KG, Selb, Germany) was used. The same samples were sintered at 1450 °C for 2 h using an ordinary furnace and a rate of temperature increase and cooling of 5 °C/min. Their density, using a He pycnometer (Micromeritics, AccupycII 1340, Norcross, GA, USA), and Vickers hardness and fracture toughness were measured using polished samples with FUTURE-TECH CORP. equipment (Milan, Italy). In the case of hardness, a load of 1 kgf was applied, which did not lead to any cracks. A higher load (5 kgf), resulting in Palmqvist cracks, was used to calculate the critical stress intensity factor values (*K_IC_*) based on the Niihara formula [22,23] proper for Palmqvist cracks:

$$K_{IC}=0.018\cdot HV^{0.6}\cdot E^{0.4}\cdot 2\cdot a\cdot l^{-0.5}$$

where *l* is the length of the crack, *a* is half of the indent, and a Young’s modulus of *E* = 200 GP was assumed. 

The biaxial flexure test—a piston on a three-ball test—in accordance with ISO6872:2008(E) was applied in order to determine the strength of the samples sintered at 1450 °C for 2 h. No surface polishing was applied before the test. Loading was performed using a Zwick/Roell Z020 machine (Zwick Roell Polska Sp.zoo. Sp.k., Wrocław, Poland). 

The microstructure was characterized using a scanning electron microscope (FEG-SEM FEI Versa 3D, (Thermo Fisher Scientific, Hillsboro, OH, USA)) equipped with an Everhart–Thornley detector (ETD) and an energy dispersive X-ray spectrometer (EDS). Electron channeling contrast imaging (ECCI) was used to show the fine grain structure. When observed using ECCI, the polished surface of the ceramic material can be observed directly without additional etching. Thermal etching usually applied in the case of zirconia polycrystals at 1150 °C could not be used since in the MgO–ZrO_2_ material, this temperature is far below the eutectoid point (1408 °C [8]) and leads to the essential phase and, hence, microstructural changes.

## 3. Results

### 3.1. Powder Characteristics

The concentration measurements of MgO in the powders resulted in a 7.4 mol% and 11.5 mol%, which is lower than the 9 and 15 mol% MgO introduced to the starting salt solutions and indicates that the co-precipitation of the MgO–ZrO_2_ system using ammonia as a reagent is not a quantitative process. 

**Figure 1:**
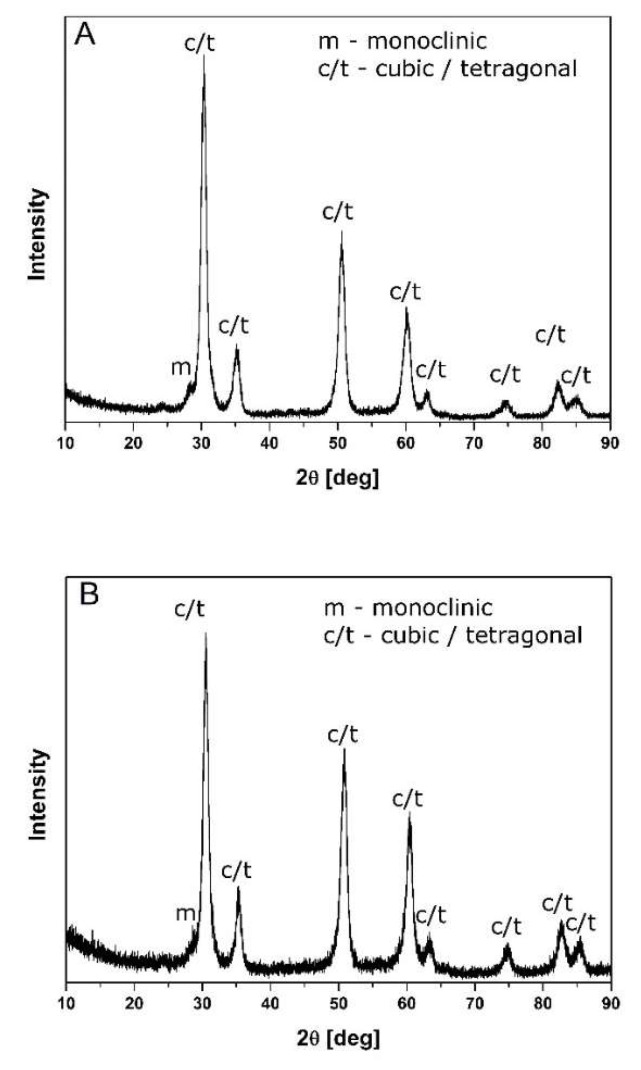
X-ray diffraction patterns of MgO–ZrO_2_ powders: (**A**) 7.4 mol% MgO; (**B**) 11.5 mol% MgO.

The data available in Table 1 suggest that the crystallites of both powders were of nanometric size. This was corroborated by the crystallite sizes (D_111_) assessed on the basis of the X-ray line broadening (111) and the results of the specific surface area measurements (S_w_). The cubic or tetragonal symmetry phases were the majority constituents of these powders (Figure 1). Due to the small crystallite sizes and the diffused large X-ray line broadening, it was not possible to distinguish these phases. 

In the generally accepted model of zirconia solid solutions, solute cations substitute zirconia atoms in the solid solution [24]. In the case of MgO–ZrO_2_ solid solutions, each Mg atom introduced into the system leads to the formation of one oxygen vacancy. Based on the X-ray diffraction measurements and assuming cubic symmetry, the density of these materials could be calculated (Table 1). These values and the specific surface area measurements allowed us to calculate the particle sizes using the relation:

D_BET_ = 6/(S_w_∙X-ray density)

**Figure 2:**
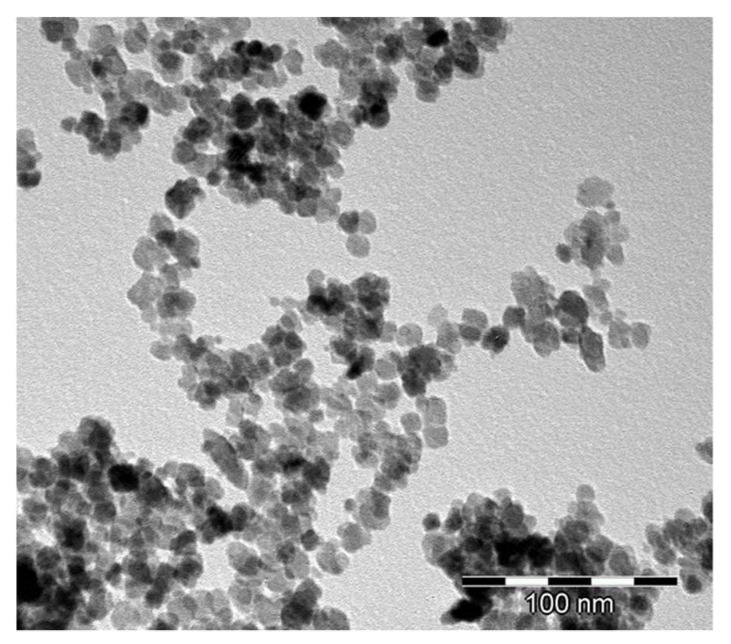
Transmission electron micrographs of the MgO–ZrO_2_ powder of 7.4 mol%.

The results shown in Table 1 show that the D_BET_ and D_111_ values were close to each other. This indicates that under the applied synthesis conditions, no essential, if any, contacts available in the BET measurements between crystallites built up. The nanometric crystallite sizes were corroborated through observation under a transmission electron microscope. This is exemplified by the TEM micrograph (Figure 2) of a powder with a lower MgO content. 

### 3.2. Compact Characteristics

**Figure 3:**
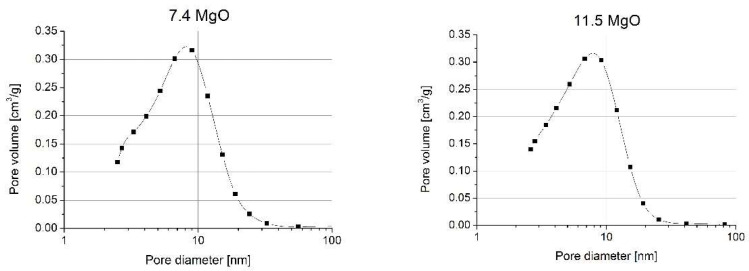
Pore size distribution (dV/dlogd) in the 7.4 mol% and 11.5 mol% MgO powder compacts.

The density of the powder compacts was determined by measuring their size and weight (see Table 2). The pore size distribution in the powder compacts shown in Figure 3 indicates their monomodal character. This, in turn, indicates that no agglomerates, eventually present in the staring powders, survived under the applied compaction pressure.

This conclusion was corroborated by the good agreement of the average pore size (dp) size calculated using the relation [25]:

$$\mathrm{dp}=D_{111}\sqrt[{3}]{\frac{V_{p}}{V_{c}}}$$

where V_p_ is the pore volume (100-relative density), V_c_ is the volume of the crystallites (relative density), and D111 is the crystallite size (see Table 1) and the modal pore size value. This should not be the case in poly-modal pore size distribution, observed in powders composed of strong agglomerates. 

### 3.3. Sintering

**Figure 4:**
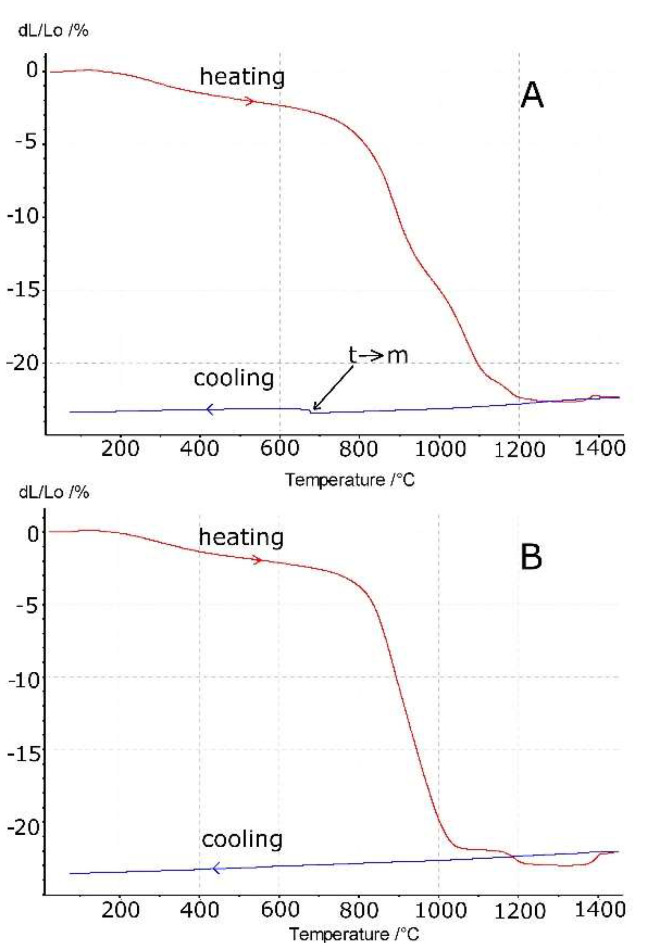
Dilatometric curves. Rate of temperature increase and decrease of 5 °C/min. The plots also show the behavior of the samples during cooling with a rate of temperature decrease of 5 °C/min. (**A**) 7.4 mol% MgO–ZrO_2_; (**B**) 11.5 mol% MgO–ZrO_2_.

Figure 4 demonstrates the shrinkage of the samples vs. temperature. The cooling runs are also shown. In both cases, we observed shrinkage which started at a rather low temperature of about 200 °C. The low temperature shrinkage is characteristic of nanometric zirconia powders and can be attributed to the desorption of water from the large surface area of the powders prepared by hydrothermal crystallization [26,27], such as those applied in the studied material (see Table 1). This effect does not occur in the compacts of the calcined powders [14] since the desorption of water takes place during the synthesis period. At an even higher temperature of about 800 °C, sintering shrinkage begins.

During the cooling period, we observed normal thermal shrinkage. Some discontinuity at about 680 °C occurred in the sample of 7.4 mol% MgO content (Figure 4A). Undoubtedly, this can be attributed to the tetragonal to monoclinic-phase transformation. A relatively high amount of the monoclinic symmetry phase occurred in this sample after cooling (Table 3). However, the material with a higher MgO content (11.5 mol%) after cooling only contained the cubic phase, which explains its monotonous thermal shrinkage (Figure 4B). Other inflexions in both materials at higher temperatures can be assigned to crystallographic-phase transformations (monoclinic to tetragonal and tetragonal to cubic).

### 3.4. Material Characteristics

**Figure 5:**
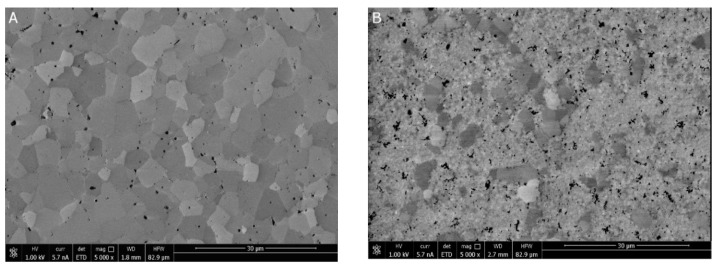

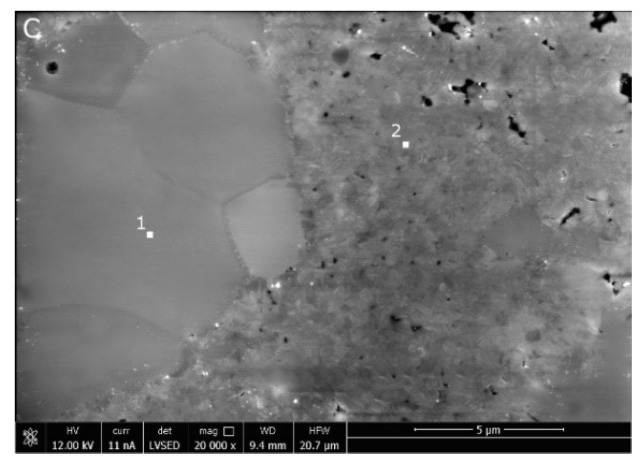
SEM images of the microstructures of the materials sintered at 1450 °C for 2 h: (**A**) Material of 11.5 mol% MgO; (**B**) Material of 7.4 mol% MgO; (**C**) The same material as B but under higher magnification. EDS analyses show 5.5 at% Mg at point 1 and 2.8 at% Mg content at point 2.

**Figure 6:**
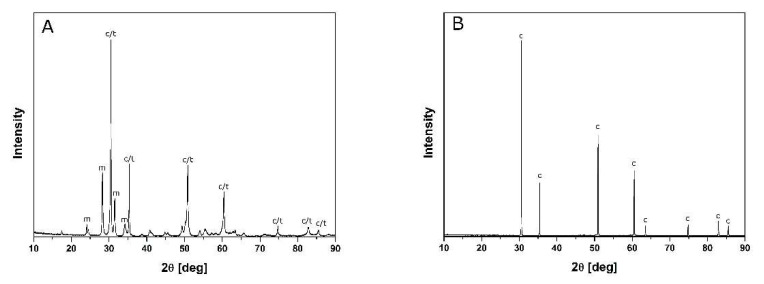
X-ray diffraction patterns of the materials sintered at 1450 °C for 2 h: (**A**) 7.4 mol% MgO; (**B**) 11.5 mol% MgO.

The pictures shown in Figure 5 demonstrate the extensive differences in the microstructures of the materials of varied MgO concentrations sintered at 1450 °C with 2 h soaking at this temperature. The material with the higher magnesia content (Figure 5A) is typical of single-phase polycrystals. The data in Table 3 and the X-ray diffraction pattern (Figure 6A) indicate the cubic symmetry of this phase, which can be explained by the fact that the chemical composition and temperature of its heat treatment corresponds to or is very close to the cubic field of the phase diagram [8]. By contrast, the system with a much lower MgO content lies in the two-phase (i.e., cubic/tetragonal) field of the MgO–ZrO_2_ system at the applied process temperature. Some of the tetragonal symmetry particles transform to monoclinic symmetry particles during cooling. The effect of this transformation is evident in the sample size increase observed during cooling (see Figure 4A). 

Figure 5B,C present the microstructure of the material with a low MgO content. The X-ray diffraction pattern (Figure 6A) and the data in Table 3 indicate the presence of cubic but also tetragonal and monoclinic phases in this material. EDS analyses at the points indicated in Figure 5C show a Mg concentration of 5.5 at.% in the grain of the larger size and 2.8 at.% in the small polycrystalline part of the sample surrounding the larger particles. This leads us to the conclusion that the larger grains correspond to the cubic symmetry phase. Thus, the matrix of the small crystallites surrounding the larger grains is composed of the monoclinic and tetragonal symmetry part of the system. Contrary to the partially stabilized zirconia manufactured in the “classical” way, no tetragonal inclusions were observed within the cubic grains [1,2,3,4,5,6,7].

The data presented in Table 3 point out the changes in the phase composition during prolonged soaking of 2 h at 1450 °C of the sample of lower MgO content compared to heat treatment with no soaking at this temperature. Undoubtedly, the mass transfer during the prolonged soaking was responsible for this phenomenon, as this leads to an increase in the cubic part of the system and the disappearance of free MgO and a decrease in the monoclinic symmetry phase. The phase composition, hardness, and fracture toughness of the studied samples sintered under the indicated conditions are shown in Table 3.

H-C means heating and cooling with no soaking time.

**Figure 7:**
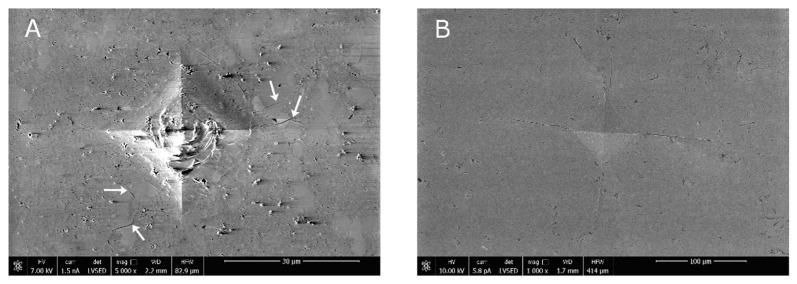
SEM micrograph of Vickers indent applied to the sample surface of (**A**) low (7.4 mol%) and (**B**) high (11.5 mol%) MgO content.

The Vickers indent, as demonstrated by the SEM micrographs (Figure 7A,B), sheds some light on the reason behind the relatively high *K_IC_* of the material with a lower MgO concentration. It seems evident that the penetration of the Vickers pyramid created numerous fractures in the material with low MgO concentration. This is seen clearly at the center of the indent. The crack deflection phenomenon (see arrows) is an additional factor that effectively participates in achieving the relatively high fracture toughness of this material. In the case of the fully cubic material, i.e., 11.5 mol% MgO, no such phenomena were detected. 

## 4. Conclusions

The performed experiments allowed us to show an innovative preparation method for nanometric powders in the MgO–ZrO_2_ system, as well as their behavior during the compaction and sintering of these powders. We identified that:

- The coprecipitation technique within the MgO–ZrO_2_ system is not quantitative when NH_3_∙aqa is used as the precipitating agent;
- Crystallization under hydrothermal conditions results in nanometric equi-axed particles with no strong contacts between them; this is due to the fact that the creation of new particles occurs in the water environment which wets and penetrates between them.
- The freeze drying of nanometric powder suspension leads to fluffy powders;
- The compacts of such powders show monomodal pore size distribution. The average pore sizes correspond well to their modal values, which indicates that agglomerates, if present in the starting powders, do not survive under compaction pressure;
- The dilatometric measurements show that the shrinkage of the compacts starts at a temperature as low as 200 °C. This was attributed to water desorption from the large surface area of the samples. Sintering shrinkage starts at about 800 °C and is completed at about 1300 °C;
- A monotonous cooling run occurs in the material with a higher MgO concentration. This is explained by its single-phase (cubic) composition. The material with a lower MgO content (7.4 mol%) shows discontinuity at about 680 °C, which can be attributed to the tetragonal- to monoclinic-phase transformation;
- The microstructure of the material with a higher MgO concentration is typical of single-phase polycrystals. However, the one with a lower MgO content shows larger cubic grains surrounded by particles of monoclinic and tetragonal symmetry. Such a microstructure differs substantially from the partially stabilized zirconia manufactured via “classical” technology. The relatively high fracture toughness of this material is explained by the formation of numerous cracks on the Vickers pyramid penetration.

## Figures and Tables

**Table 1:** Characteristics of MgO–ZrO_2_ powders.

MgO mol%	Sw m^2^/g	D_BET_ nm	D_111_ nm	Monoclinic wt%	X-ray Density g/cm^3^	Cubic/Tetragonal wt%
7.4	124.5	8.1	8.5	9.1	5.91	90.9
11.5	128.3	7.9	8.3	9.5	5.92	90.5

**Table 2:** Compact characteristics.

Powder	Density g/cm^3^	Relative Density %	Average Pore Size dp nm	Mode, nm
7.4 mol% MgO	2.59	43.8	9.2	9.0
11.5 mol% MgO	2.58	43.6	9.0	9.0

**Table 3:** Phase composition, hardness (*HV*), critical stress intensity factor (*K_IC_*), strength (σ), and density (*d*).

Sample Sintering Conditions	Monoclinic wt%	Tetragonal w%	Cubic wt%	MgO wt%	*HV*, GPa	*K_IC_*, MPa∙m^1/2^	σ, MPa	*d*, g/cm^3^
7.4 mol% MgO, 1450°C, (H-C)	72.2	14.4	11.5	1.9	9.4 ± 0.8	9.48 ± 0.20	-	5.581 ± 0.003
7.4 mol% MgO, 1450°C, 2h	41.6	13.8	44.6	-	10.5 ± 0.3	8.75 ± 0.18	424 ± 3	5.679 ± 0.003
11.5 mol% MgO, 1450 °C, (H-C)	-	-	100	-	11.4 ± 0.6	5.77 ± 0.14	-	5.395 ± 0.002
11.5 mol% MgO 1450 °C, 2h	-	-	100	-	10.9 ± 0.7	5.29 ± 0.11	198 ± 4	5.541 ± 0.002

## Data Availability

The data presented in this study are available on request from the corresponding authors.
